# Surface Coatings of Dental Implants: A Review

**DOI:** 10.3390/jfb14050287

**Published:** 2023-05-22

**Authors:** Angelo Michele Inchingolo, Giuseppina Malcangi, Laura Ferrante, Gaetano Del Vecchio, Fabio Viapiano, Alessio Danilo Inchingolo, Antonio Mancini, Ciro Annicchiarico, Francesco Inchingolo, Gianna Dipalma, Elio Minetti, Andrea Palermo, Assunta Patano

**Affiliations:** 1 Department of Interdisciplinary Medicine, University of Bari “Aldo Moro”, 70124 Bari, Italy; angeloinchingolo@gmail.com (A.M.I.); giuseppinamalcangi@libero.it (G.M.); lauraferrante79@virgilio.it (L.F.); dr.gdelvecchio@gmail.com (G.D.V.); viapianofabio96@gmail.com (F.V.); ad.inchingolo@libero.it (A.D.I.); dr.antonio.mancini@gmail.com (A.M.); annicchiarico.ciro63@gmail.com (C.A.); assuntapatano@gmail.com (A.P.); 2 Department of Biomedical, Surgical, and Dental Science, University of Milan, 20122 Milan, Italy; elio.minetti@gmail.com; 3 College of Medicine and Dentistry Birmingham, University of Birmingham, Birmingham B4 6BN, UK; andrea.palermo2004@libero.it

**Keywords:** osseointegration, surface, coating, dental implant, titanium, treatment surface, peri-implant health, implant stability, bacterial adhesion, marginal bone level

## Abstract

Replacement of missing teeth is possible using biocompatible devices such as endosseous implants. This study aims to analyze and recognize the best characteristics of different implant surfaces that ensure good peri-implant tissue healing and thus clinical success over time. The present review was performed on the recent literature concerning endosseous implants made of titanium, a material most frequently used because of its mechanical, physical, and chemical characteristics. Thanks to its low bioactivity, titanium exhibits slow osseointegration. Implant surfaces are treated so that cells do not reject the surface as a foreign material and accept it as fully biocompatible. Analysis of different types of implant surface coatings was performed in order to identify ideal surfaces that improve osseointegration, epithelial attachment to the implant site, and overall peri-implant health. This study shows that the implant surface, with different adhesion, proliferation, and spreading capabilities of osteoblastic and epithelial cells, influences the cells involved in anchorage. Implant surfaces must have antibacterial capabilities to prevent peri-implant disease. Research still needs to improve implant material to minimize clinical failure.

## 1. Introduction

Natural tooth loss has serious emotional, psychological, and social effects in addition to physical and functional effects on an individual [1]. Implantoloy is one of the most secure and effective surgical procedures [2]. The most common dental implant materials are titanium, zirconium, and polyetheretherketone (PEEK) [3,4].

Zirconium implants have good aesthetic qualities but a moderate rate of fracture, which leads to implant failure [5]. PEEK implants have demonstrated high fallibility rates; hence, long-term multicentric studies are required to confirm the reliability [6].

Titanium is the material that best complies with the requirements of dental implantology, including osseointegration, biocompatibility, mechanical resistance, and anti-bacterial properties [7,8]. The term “osseointegration” was first used by Albrektsson (1981) to refer to the functional and structural connection between a vulnerable structure’s surface and its critical organs [9]. Accordingly, a number of critical factors for proper bone resorption have been identified: biocompatibility, implant design, implant surface characteristics, condition of the recipient bone site, surgical technique, operator’s skill, and implant storage conditions [9].

The characteristics of the implants’ surface and the quality of the recipient site bone determine the interface between the two: the bone–implant interface [10]. For instance, an implant positioned in a lamellar bone has 90% contact, whereas one positioned in a midollar bone has 50% contact [11,12].

Morphologically, dental and implant periodontal tissues have many common features, as both are marked by a well-keratinized oral epithelium and a portion of connective tissue in direct contact with the implant and tooth [13]. More collagen and fewer fibroblasts are found in the implantable connective tissue [13,14].

The physicochemical properties of the implant outermost layer and its interaction with the surrounding essential tissues play a role in determining whether osseointegration succeeds or fails [15].

A fundamental prerequisite for the long-term success of the implant is biological anchorage between the surface of the dental implant and the bone tissue [16,17]. Bone response is closely related to the implant surface [17].

**Figure 1:**
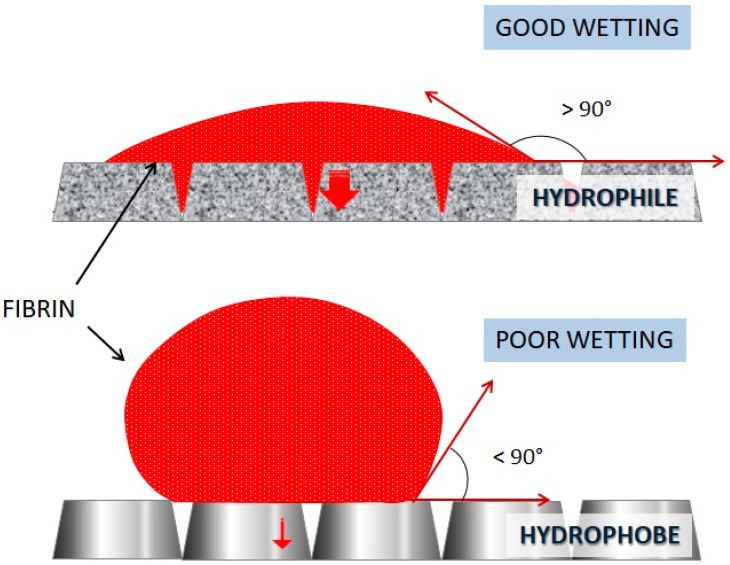
Fibrin implant wettability. The hydrophobe surface shows poor wettability, unlike the hydrophile one with good wettability. The red arrow indicates the magnitude of liquid permeability on the surface of the implant.

Hydrophilic and hydrophobic implant surfaces can be distinguished [11,18]. Hydrophilic surfaces, compared with hydrophobic structures, favor interactions with biological fluids and cells allowing a good surface wettability [18,19]. Implant surfaces with the same chemical composition actually offer a different contact angle for biological fluids depending on the topography of the surface: rough surfaces, such as sandblasted and etched surfaces, are more likely to be wettable than surfaces considered to be smooth [18,20] (Figure 1).

**Figure 2:**
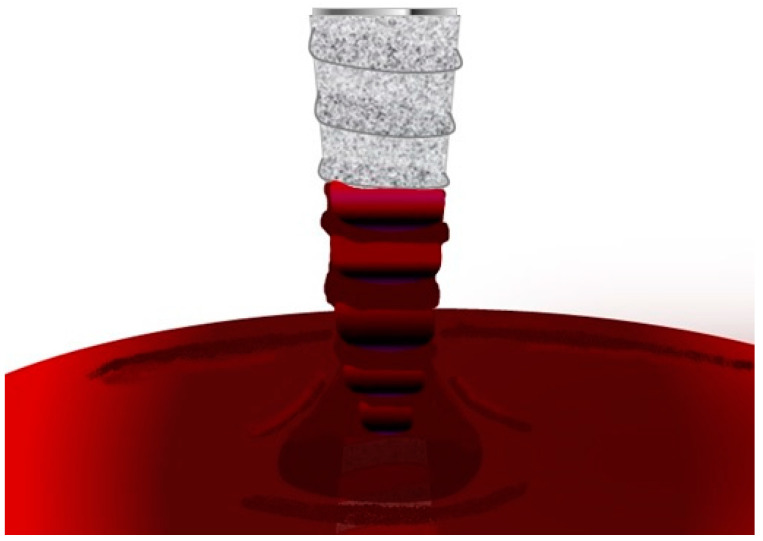
Good implant wettability: As soon as the implant is inserted into the bone, there is immediate blood–fixture contact. Blood is attracted to the implant surface.

The hydrophilicity of the implant surface results in abrupt contact of the implant with the clot, favouring the osseointegration process [21,22]. Some surfaces have such hydrophilicity that mere contact of the first coils with blood results in suction along the entire implant surface [21,21] (Figure 2). Roughened surfaces increase blood clot retention [23].

**Figure 3:**
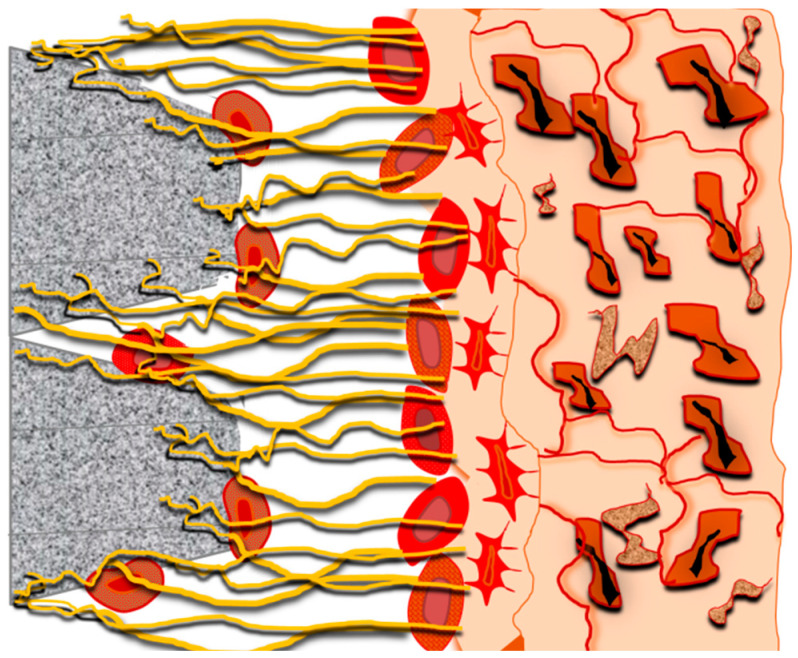
Fibrin adhesion to the implant surface.

The implant surface treated with rumination exhibits a double retraction of fibrin filaments and a double blood clotting compared with the smooth surface [24] (Figure 3).

During the wound healing phase, the following takes place:

- Fibrin formation that protects the wound and, together with platelets, plugs the wound and releases the repair factors;
- Fibrinolysis: reabsorption of the clot;
- Osteoclastic activity: migration of cells from the blood;
- Migration of mesenchymal cells, precursors of bone cells [25,26,27].

Implant stability is necessary for effective osseointegration and healing [3,13,24,28]. The features of the bone, the implant’s design, and the procedure used to place it all affect primary stability [29]. Bone remodeling and bone production around the implant lead to secondary stability [30]. Growing research demonstrates that implant surface features also affect secondary stability [31,32].

Bone apposition on the implant surface begins first in trabecular bone, then in compact bone [24,33]. Peri-implant bone metabolism is at its peak 1–4 months after surgery [34,35].

**Figure 4:**
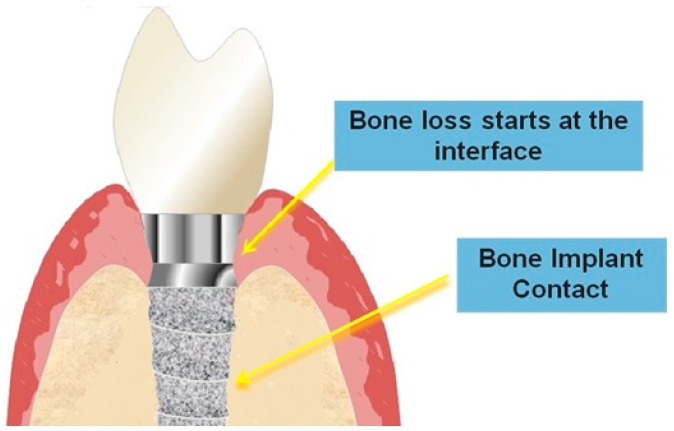
Schematic image of the implant–bone interface.

The clinical success of the implant, in addition to osseointegration, depends on the health of the bone–implant–soft tissue interface [16] (Figure 4).

Implant failure may result from titanium’s reduced ability to induce osseointegration, which causes poor or delayed osseointegration [36,37]. Furthermore, early titanium implants had a mechanically polished surface that was smooth, and research in recent years revealed that this surface is less stable over time than those with a rough surface [36,38]. In order to achieve a larger contact surface, treatment of the implant surface was performed in order to increase the osseointegration between the bone and the implant [36].

This review aimed to analyze different surfaces and, therefore, identify the ideal implant structure from a clinical and durability point of view, with the least post-surgical complications and the least discomfort to the body [17,39,40]. Research is extensive and challenging because of ongoing scientific discoveries and innovations [41].

In fact, an appropriate modification of titanium surface, which increases the percentage of BIC (bone implant contact), is still being studied to favor osseointegration, which has antibacterial properties to prevent peri-implant diseases and resists the stresses it will undergo with functionalization, such as chewing, thus guaranteeing healthy peri-implant tissue over time [30,39,42].

## 2. Materials and Methods

### 2.1. Search Processing

The present review was performed in accordance with the principles of PRISMA. PubMed, Scopus, and Web of Science were searched to find papers that matched our topic dating from 1 January 2019 up to 31 March 2023, with English-language restriction. The search strategy was built using a combination of words that matched the purpose of the investigation, whose primary focus is the difference of implant surface coatings on osseointegration; hence, the following Boolean keywords were used: different dental implant surface AND osseointegration (Table 1).

### 2.2. Inclusion and Exclusion Criteria

The inclusion criteria were as follows: (1) human in vivo study; (2) English language; (3) open access studies; (4) clinical studies; (5) studies examining the variety of surfaces of titanium dental implants: implant surface treatments and coatings; and (6) in vitro studies concerning the analysis of implant surface coatings of great interest to our research.

The exclusion criteria were as follows: (1) animal; (2) other languages different from English; (3) not open access studies; (4) case report/series, reviews, editorials, book chapters; (5) research about zirconium and peek dental implant; and (6) in vitro studies far from the focus of our research.

The review was conducted using the PICO criteria:

- Population: Titanium endosseous implants;
- Intervention: Implant surface treatment;
- Comparisons: Different implant surfaces;
- Outcomes: Interaction with biological tissues;

### 2.3. Data Processing

Author disagreements on the choice of articles were discussed and settled.

## 3. Results

**Figure 5:**
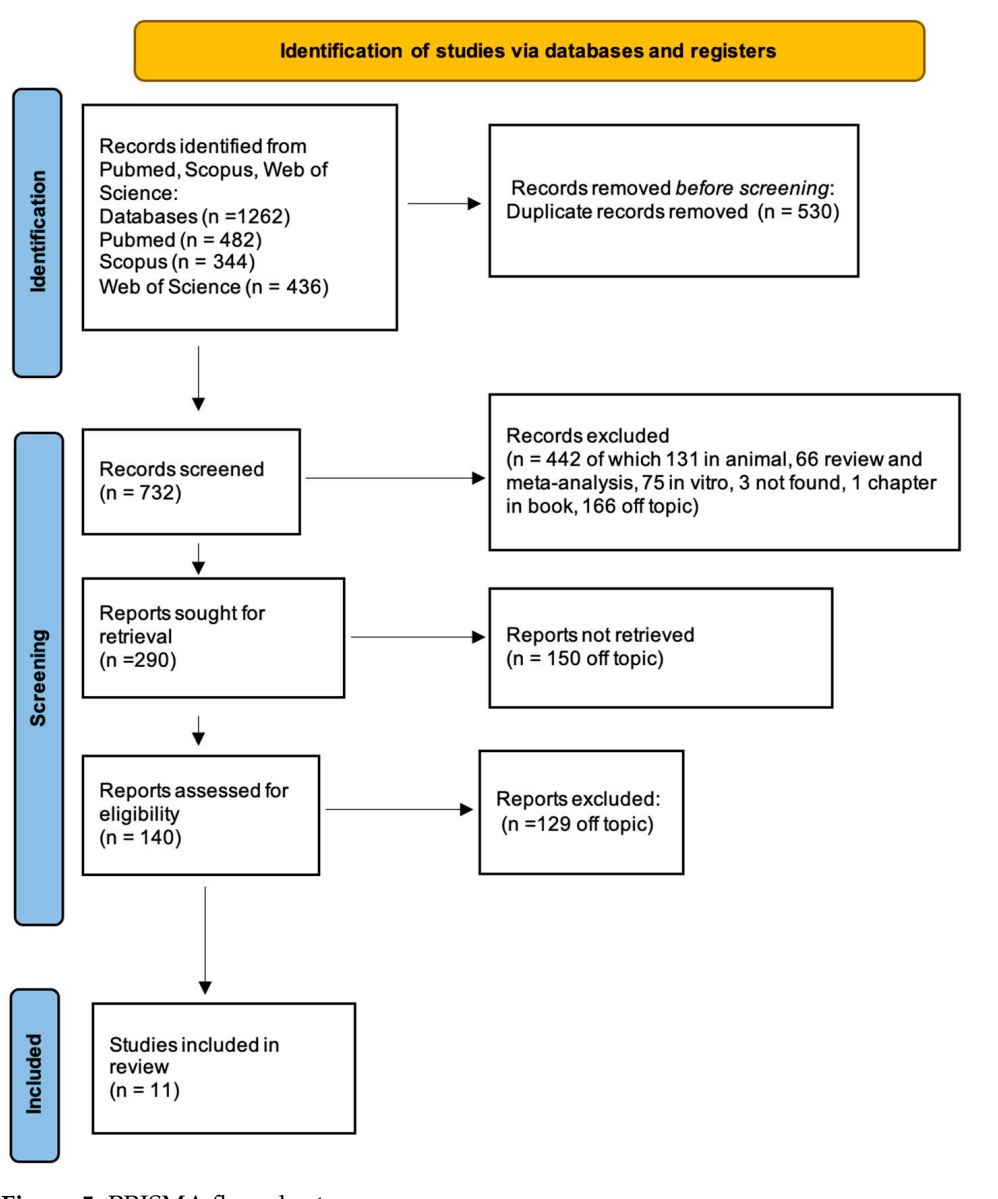
PRISMA flow chart.

A total of 1262 publications were identified from the following databases, Pubmed (482), Scopus (344), and Web of Science (436), which led to 732 articles after removing duplicates (530). A total of 290 articles accessed the screening phase, while 442 items were removed because 3 were not found, 131 were in animal, 1 was a chapter in a book, 75 were not in vivo and far from the focus of this review, 66 were reviews and meta-analyses, and 166 were off topic. From these papers, 279 were additionally removed because of lack of interest and eligibility was assigned to 11 records that were finally included in the review for qualitative analysis, of which 5 were in vitro (Figure 5). The results of each study are reported in Table 2 and Table 3.

## 4. Discussion

Thanks to its excellent mechanical properties, including biocompatibility, corrosion resistance, non-magnetism, and non-toxicity, titanium and its alloys are widely used to create body armor and dental implants, with success rates close to 95–97% [51].

It is also very reactive and forms an ossidic layer of about 5 nm in thickness, which, in contact with air and water, protects it from corrosion and improves its affinity for patient cells [8].

However, even if titanium is a biologically inert material, it lacks anti-bacterial properties [52]. As a result, bacteria tend to adhere to the collars of implants, and implant failure can be linked to peri-implant infections [51,53]. Once discovered, perimplantite must be treated with antibiotics, which not only increases the risk of developing antibiotic resistance but also causes discomfort and costs the patient money [54]. As a result, it is crucial that titanium implants have long-term anti-bacterial properties and improve early osseointegration capability [55,56]. To meet these clinical requirements, it is necessary to apply a treatment to change the surface of pure titanium, optimizing the surface’s morphology and chemical composition [8].

Researchers are working to increase the capacity of the surfaces of titanium machinery [57]. The surface, shape, and structure of the implant affect the osseointegration process, which is necessary to provide implant stability [36]. The stability of the implant, both primary and secondary, is a factor that affects how well the implant itself will osseointegrate [58]. While the primary stability is a mechanical phenomenon that depends on both the implant’s macroscopic and microscopic design and the surgical technique used to position it, numerous studies have found that the implant’s surface is the key factor in achieving a high level of secondary stability [58,59].

Among the characteristics of implant surfaces, topography and chemical composition are those that have the most impact on the interaction between biomaterial and osseous tissue and, consequently, on secondary stability [17,29]. In particular, numerous studies have demonstrated that, compared with implant surfaces, textured surfaces exhibit a greater capacity for determining a biological response from some osseous cellular lines [60]. In fact, the roughness provides a larger area of contact and interconnection, leading to a greater number of cellular colonies that create strong adhesions to the implant site and enhancing osteoblast proliferation and adhesion processes while decreasing osteoclastic activity and promoting mineralization [61]. In addition, implant roughness aids in the differentiation of mesenchymal cells into the osteoblastic phenotype [29].

Physicochemical treatments of major implant surfaces give rise to different types of implants:

- machined;
- polished;
- treated;
- hybrid [17,62].

**Figure 6:**
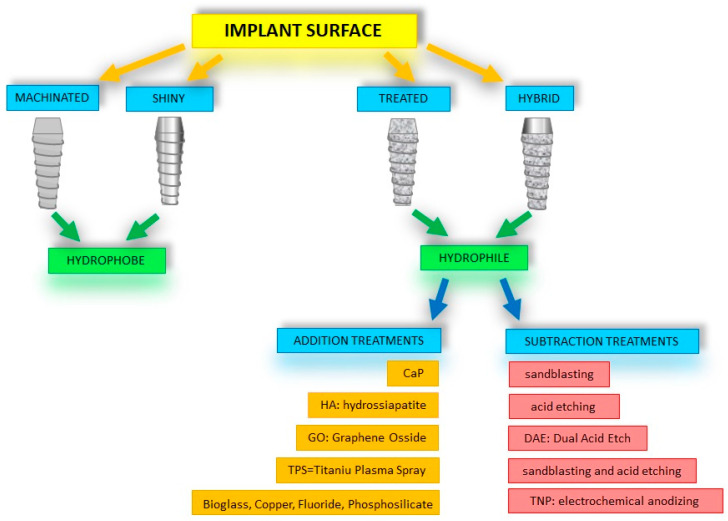
Summary diagram of implant surface treatments. The diagram shows how the hydrophilic property of the implant surfaces (treated and hybrid) lends itself better than the hydrophobic surfaces (machined and smooth) to further treatments to improve their general characteristics.

A significant advantage of treated and hybridized surfaces is the increased degree of hydrophilicity and wettability compared with untreated, machined, smooth surfaces, which are considered hydrophobic [19]. The only way to modify something on the surface is to add or reduce materials on a micro- or nanometric scale [50] (Figure 6).

**Figure 7:**
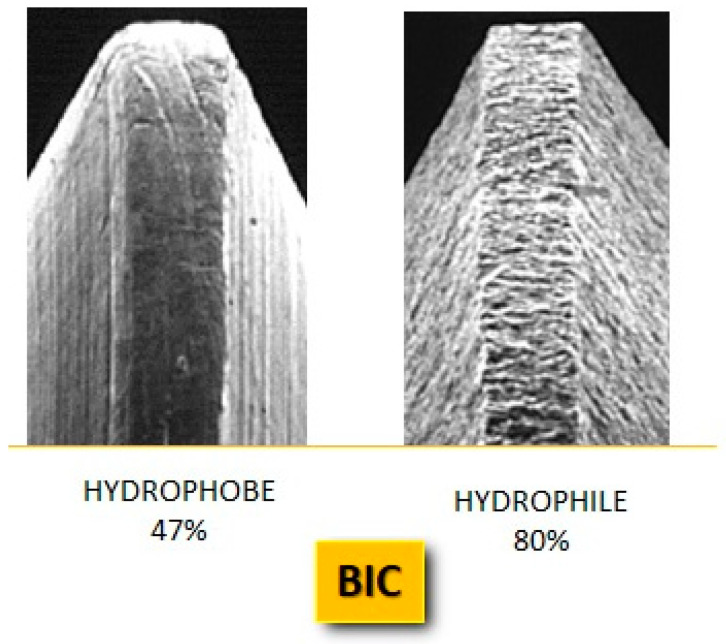
Electron microscopy detail of a smooth (hydrophobe) and a rough (hydrophile) implant surface, with re-wetting percentages. The hydrophilic surface has a higher BIC percentage than the hydrophobic surface.

Bone-to-implant contact (BIC), early in the healing phase, is considerably increased by implants with a hydrophilic surface because these implants typically display more cell differentiation and aggregation [11] (Figure 7). 

The most significant advancement in implant dentistry has been the observation of direct bone-to-implant contact (BIC), which was verified with electron microscopy [47].

In comparison with freshly worked surfaces, titanium dental implants with moderately rough surfaces exhibit better osseointegration and faster osseous growth [50].

### 4.1. Implant Surface Treatments

#### 4.1.1. Subtraction Treatments

A technique for creating moderately rough implant surfaces is sandblasting and acid mordantation (SA) [50,60,63]. According to some studies, the surface modification using the SA technique needs to be properly planned and managed in order to produce a final medical device that is clean and reliable [17]. This is because it has been observed that the majority of implant surface areas contain particulates, which are remnants of the sandblasting [64]. This causes a 15% reduction in tensile strength, which could lead to the beginning of a fracture process [9,11,17,65].

On the other hand, for the past ten years, a widely employed method of surface modification has been the combination of sandblasting and etching [66]. Sandblasting theoretically allows to achieve the ideal roughness for mechanical fixation, while additional etching, by raising the peak height of the roughness peaks, allows to enhance the protein adhesion mechanism, which is crucial in the early stages of bone healing [67]. In fact, these two techniques are used in succession [48].

Surface alteration techniques that use subtractive processes include sandblasting and acid etching [62]. Acid etching causes selective corrosion to occur, leaving holes or grooves on the metal surface [38,68].

Because of its hydrophilic qualities, sandblasted, coarse-grained, acid-etched (SLA) surface is a characteristic form of rough surface generated on a dental implant and has been employed on the newest commercial dental implants [68].

Dual Acid Etch, or DAE Technology, uses double acid etching without first sandblasting [69]. Using this method, the danger of ingesting sand particles is reduced, and surfaces are created that improve BIC, platelet retention, and the release of bone growth hormones [70,71,72].

By producing a special titanium surface with distinctive meso, micro, and nanoscale roughness features that ensure better osteoconductive and osseointegrative capacities than the more popular micro-rough titanium surface, a method for enhancing osseointegration has been devised [45]. Sulfuric acid was used to etch commercially pure titanium at four different temperatures (120, 130, 140, and 150 °C) [45]. Particularly when acid etching was carried out at 140 °C, the new surface considerably stimulated osteoblast development and, subsequently, osseointegration [17,45].

One of the nanoengineering methods for titanium implants is called electrochemical anodizing [73]. This method involves immersing the titanium implant, which serves as the anode, in an organic electrolyte containing water and fluoride in an electrochemical cell with appropriate voltage, such that titania nanopores (TNPs) are created on the implant surface in order to enhance soft tissue integration and wound healing [73,74]. Anodizing has emerged as a useful technique for changing the surface morphology of titanium or titanium alloys to enhance bone development because it is inexpensive, simple to apply, and easy to control [75,76]. Anodizing can provide a surface morphology with a pore structure on a micronano scale as well as increase the wear and corrosion resistance of pure titanium implants [8].

Further frontiers of research that deserve further investigation are 3D-printed implants and micro-ark oxidizing, which help improve biocompatibility. These are promising fields that will offer new possibilities in the future of clinical practice [77].

#### 4.1.2. Addition Treatments

Biomaterials in implantology have been promoting bone response and biomechanical ability in recent years [78,79]. Many substances, including polyhydroxyalkanoates, calcium phosphate, carbon, bisphosphonates, hydroxyapatite, bone-stimulating agents, bioactive glass, bioactive ceramics, collagen, chitosan, metal and their alloys, fluoride, and titanium/titanium nitride, are known as promising candidates for dental implant coatings [78,80]. It is crucial that biomaterials degrade naturally; polyhydroxyalkanoates, for instance, degrade naturally and do not harm tissues or cells in the process [81,82].

Owing to the development of biofilms, which are thought to aid bacteria in evading antibiotics and the host defense mechanism, bacterial colonization of titanium results in implant loss. Pathogens cause deterioration of the bone surrounding the implant, necessitating surgery to repair the infected bone or to remove or replace infected implants. [37,78,83,84].

Although both implant types generated comparable clinical outcomes at 12 weeks following surgery, implants with alkali-modified surfaces were consistently more stable after implantation than implants with sandblasted surfaces [44,85].

Improved contact osteogenesis surrounding the dental implant was seen on surfaces coated with calcium phosphate (CaP), and early healing phase osseointegration was also seen to be enhanced [9,36]. Increasing the biocompatibility of titanium and encouraging osteogenesis were among the first goals to be achieved by researchers, and for this, some authors employed chemical modifications, such as the addition of fluoride to the implant surface [48].

The interaction of fluoride with hydroxyapatite in bone tissue creates fluorapatite followed by increased osteoblast proliferation and activation of alkaline phosphatase activity [86,87,88]. Because of its outstanding physical and chemical characteristics, particularly its potential for osteoinduction, graphene oxide (GO) is a promising nanomaterial [57,89,90]. The addition of inorganic bioactive elements confers the important and necessary osteogenic, angiogenic, and antibacterial capabilities [53].

Broad-spectrum antibacterial capabilities, high efficiency, and durability are all properties of copper (Cu) [86,91]. Copper-containing titanium alloy has been confirmed to have a constant precipitation of copper ions and long-lasting antibacterial activity [53,83,92]. It is a necessary trace element for the human body because it can prevent osteoporosis, promote osteogenic differentiation, and induce angiogenesis [93]. Ti-5Cu alloy has remarkable anti-infective efficacy, osteogenic potential, and biological compatibility, which have been amply demonstrated by laboratory investigations [8,91,94].

Implants are frequently vulnerable to infections like peri-implantitis, which affect the surrounding hard and soft tissues and result in implant loss and biocompatibility [95].

**Figure 8:**
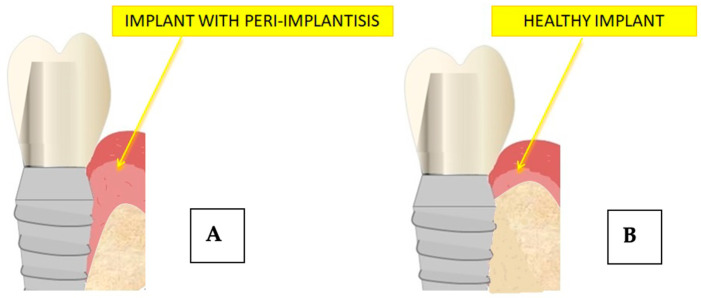
Difference in implant bone contact in conditions of implant good health (**B**) and during peri-implantitis disease (**A**).

Peri-implantitis is an inflammatory condition that affects all surrounding tissues [96]. A deep pocket with hemorrhage, suppuration, and slight bone loss accompanies mucosal injury [33,97,98] (Figure 8).

Therefore, a recent scientific study has focused on the interface between the implant and the surrounding soft tissues, highlighting the significance of establishing a sufficient epithelial biological seal that is necessary to prevent bacterial contamination [17,97,99,100]. The underlying bone tissues are shielded from germs by the peri-implant tissues, which are made up of connective and epithelial components [101]. It has been claimed that coating the implant with bioactive materials will help to avoid the development of this disease [49].

A bioactive glass known as Bioglass 45S5 or calcium sodium phosphosilicate is made up of silica, calcium oxide, phosphorus pentoxide, and sodium oxide [102]. Biomaterials for bone grafts, periodontal defect repair, cranial and maxillofacial repair, wound care, blood loss management, stimulation of vascular regeneration, and nerve repair are among the typical uses of Bioglass 45S5 [49].

#### 4.1.3. CGF Coated Dental Implants

More recent studies are focusing on the biological properties of growth factor concentrate (CGF), an autologous blood-derived biomaterial, in improving the osseointegration of dental implants [103,104]. The surface of CGF permeated dental implants is biocompatible and biologically active, significantly improving the adhesion of endothelial cells to the implants themselves [31]. All of this guarantees better results in terms of osseointegration and decline in post-surgical complications [31,103].

Some basic parameters are to be monitored during the osseointegration period and after loading to ascertain peri-implant health over time: early healing index, visible plaque index, tartar, peri-implant inflammation, probing depth and bleeding at probing, implant stability quotient, crestal bone loss, bone level variation, and implant success and survival rates [105]. Implant surface modifications can improve implant durability and health and thus ensure proper prosthetic rehabilitation [38,106]. This is also especially true in those patients in whom implant-prosthetic rehabilitation is not only cosmetic, but functional [107]. Sometimes, such patients have systemic diseases or have compromised bone conditions [108]. Implants with surface treatments that can improve bone–implant interactions, protein uptake, adhesion, differentiation, and cell proliferation have been used in these patients. In clinical trials with patients using anticoagulants, diabetics, people who had radiation therapy to the head and neck, and people who needed bone grafting, implants with hydrophilic surfaces displayed encouraging outcomes [109,110]. In comparison with other treated, coated implants, mandibular implant overdentures showed considerably higher 1-year survival rates in clinical trials using SLActive hydrophilic surfaces [43,110,111].

## 5. Conclusions

From the present study, it emerged that, although all surfaces allowed osseointegration and cell proliferation, the treated surfaces, owing to surface irradiation, had a better propensity for epithelial cell attachment and adhesion, proliferation, and differentiation of osteoblastic cells.

However, research must be directed not only to the osseointegration of the implant into the bone structure, to ensure primary and secondary stability, but also to the seal that the soft tissues provide superficially, which is essential to protect the peri-implant tissues and stability of the prosthesis.

Researchers in designing an implant must give equal importance to both osseointegration and mucointegration, key parameters for generating stability and creating a mucosal seal around the prosthesis. Research in micro and macro implant topography must be focused on designing successful medical devices, reducing clinical failure.

In summary, the primary objectives for the creation of implant surface changes are as follows:

- Enhance clinical effectiveness in regions with both qualitative and quantitative bone deficiencies;
- Speed up the osseointegration process so that immediate or early loading protocols can be addressed;
- Sncourage bone formation in areas where there is insufficient alveolar ridge to enable the implantation of implants;
- Properly seal the muco-gingival biological junction in order to prevent bacterial contamination.

Owing to continuous scientific discoveries and innovation, research is extensive and expanding. It is difficult to apply research in vivo; therefore, a long period of control is still necessary before being able to have certain results on patients.

## Figures and Tables

**Table 1:** Database search indicators.

Articles’ Screening Strategy
KEYWORDS: A: different dental implant surface; B: osseointegration.
Boolean Indicators: A AND B.
Timespan: 2019–2023.
Electronic databases: Pubmed; Scopus; WOS.

**Table 2:** Characteristics of the in vivo studies included in the qualitative analysis.

Authors (Year)	Type of the Study	Aim of the Study	Materials	Results
Bielemann et al. (2022) [43]	Randomized controlled trial	Evaluate the clinical and radiological peri-implant parameters between hydrophilic and hydrophobic dental implants	For 2 types of surfaces, hydrophobic and hydrophilic, different peri-implant health indices were evaluated: (i) early healing index (EHI), visible plaque index (VPI), presence of tartar (CP), peri-implant inflammation (PI), probing depth (PD), and bleeding on probing (BOP); implant stability quotient (ISQ), crestal bone loss (CBL), and bone level change (BLC); and implant success and survival rates.	There were no differences in peri-implant healing, stability, and bone remodeling after 1 year.
Gursaytrak et al. (2020) [44]	Randomized controlled trial	Evaluate the stability of implants with different surfaces (alkali-modified or sandblasted) using resonance frequency analysis (RFA).	Immediately after implantation as well as at 2, 6, and 12 weeks, RFA was utilized to assess the stability quotient of implants with alkali-modified (bioactive) and sandblasted surfaces.	After placement, implants with alkali-modified surfaces were more stable than implants with sandblasted surfaces after, but the two types had similar clinical results at 12 weeks after surgery.
Hasegawa et al. (2020) [45]	Randomized controlled trial	Optimize the implant surface’s biological potential for improved osseointegration.	The titanium surface was etched with sulfuric acid at different temperatures (120, 130, 140, and 150 °C).	The maximum capacity for osseous integration was reached when the surface of the implant was acidified at 140 °C, significantly increasing the capacity for osteoconductive and osteointegrative growth.
Ko et al. (2019) [46]	Randomized controlled trial	Comparing the peri-implant marginal bone level around CaP-coated and uncoated sandblasted, large-grit, acid-etched (SLA) surface implants 1 year after implantation.	Clinical and radiographic examinations were performed to assess initial stability and changes in marginal bone level after 3 months and after 12 months.	All of the implants were successful.
Kormoczi et al. (2021) [47]	Randomized controlled trial	Comparison of early loaded implants with different modified surface stability.	Implant success, implant stability, and periodontal parameters were evaluated after the placement of implants with SA (alumina blasting and acid etching), NH (bioabsorbable apatite nanocoating), or SLA (coarse-grain blasting and acid etching) surfaces.	No significant differences were found in the two groups and good periodontal parameters were found.
Velloso et al. (2019) [11]	Randomized controlled trial	Evaluating the effects of implant devices with the same brand, design, length, and diameter but with two different surface treatments: sandblasting and etching with acid (SAE) and SAE modified chemically (hydrophilic).	20 distinct patients received 20 implants with the same shape, size, and diameter but with two different surface treatments (10 SAE and 10 modified SAE). After six weeks, implant stability values were assessed.	Implants with a modified SAE surface showed superior and faster implant stability.

**Table 3:** Characteristics of the in vitro studies included in the qualitative analysis.

Authors (Year)	Type of the Study	Aim of the Study	Materials	Results
Chauhan et al. (2021) [48]	In vitro	To investigate the action of acid etching on the surface characteristics of titanium alloy implants and to optimize the process variables to produce micro- and nanotopography on the surface of dental implants.	Without heating the acid solution, the optimum implant surface was carefully examined and compared with the etched surface.	Titanium alloy had a very different surface topography than commercially pure titanium, and it had a distinct surface topography depending on whether the attachment was done at ambient temperature or at higher temperature, which has an impact on cells’ behavior
Gavinho et al. (2019) [49]	In vitro	Analyze Bioglass 45S5 with CeO, evaluating whether its antioxidant effect reverses oxidative stress after implantation in bone.	The materials’ morphological, structural, and biological properties (cytotoxicity, bioactivity, and antibacterial activity) were examined.	The addition of cerio did not lead to structural changes in the biocompatible glass, which did not exhibit cytotoxicity, but it prevent the growth of Escherichia coli and Streptococcus mutans, and all of the tests revealed the initial deposition of a CaP-rich layer on the material’s surface after 24 h.
Rausch et al. (2021) [17]	In vitro	Evaluate the ability of human gingival cells to attach to and grow on differently treated titanium or zirconia implant surfaces	Zirconia and titanium implant surfaces were treated differently and subsequently had different roughness: some surfaces were machined and smooth, while other surfaces were sandblasted and rough.	Gingival cell behavior is mainly influenced by surface roughness, and no relevant difference was found between titanium and zirconia implants.
Schupbach et al. (2019) [50]	In vitro	Comparing several commercially available implant systems with SA-modified surfaces and their surface-level morphological and cleaning characteristics.	Six candidates from three different lots were chosen to be the installation team for each system. The average particulate counts for each project were calculated from three different interest regions and compared.	Not all manufacturers can create implant surfaces without contaminating them with particulates.
Zhang et al. (2021) [8]	In vitro	Reduce associated infection symptoms and improve early osseointegration of dental implant.	Anodic oxidation with hydrogen fluoride was performed on the Ti-Cu alloy implant surface.	Etching hydrogen fluoride + Ti-5Cu alloy revealed that it has high corrosion resistance, great biological compatibility, and extremely potent antibacterial characteristics.

## Data Availability

Not applicable.

## Abbreviations

BIC	Bone implant contact
BMMSC	Multipotent mesenchymal stem cells from bone marrow
CaP	Calcium phosphate
CGF	Concentrated growth factors
Cu	Copper
DAE	Dual acid etch
GO	Graphene oxide
HF	Hydrogen fluoride
PEEK	Polyetheretherketone
RFA	Resonance frequency analysis
SA	Sandblasting and acid etching
SEM	Scanning electron microscopy
SLA	Coarse-grain blasting and acid etching
Ti	Titanium
Ti-5Cu	Titanium-copper alloy
TPS	Titanium plasma spray
TNP	Titanium nano pores
